# Identification of *Brachypodium distachyon B3* genes reveals that *BdB3-54* regulates primary root growth

**DOI:** 10.3389/fpls.2022.1050171

**Published:** 2022-11-10

**Authors:** Jie Guo, Hanxiao Liu, Keli Dai, Xiangyang Yuan, Pingyi Guo, Weiping Shi, Meixue Zhou

**Affiliations:** ^1^ College of Agronomy, Shanxi Agricultural University, Jinzhong, China; ^2^ Tasmanian Institute of Agriculture, University of Tasmania, Prospect, TAS, Australia

**Keywords:** B3 transcription factor, *Brachypodium distachyon*, genome-wide, root development, transgenesis

## Abstract

B3 is a class of plant-specific transcription factors with important roles in plant development and growth. Here, we identified 69 B3 transcription factors in *Brachypodium distachyon* that were unevenly distributed across all five chromosomes. The ARF, REM, LAV, and RAV subfamilies were grouped based on sequence characteristics and phylogenetic relationships. The phylogenetically related members in the B3 family shared conserved domains and gene structures. Expression profiles showed that *B3* genes were widely expressed in different tissues and varied in response to different abiotic stresses. *BdB3-54* protein from the REM subfamily was located in the nucleus by subcellular localization and processed transcriptional activation activity. Overexpression of *BdB3-54* in Arabidopsis increased primary root length. Our study provides a basis for further research on the functions of *BdB3* genes.

## Introduction

B3 transcription factors (TFs) are plant-specific and contain at least one B3 domain. The B3 domain is named according to its position in the third basic region of the maize (*Zea mays* L.) VIVIPAROUS-1 protein (Suzuki et al., 1997). This B3 domain of approximately 110 amino acids forms two short α-helices and seven β-barrels (Swaminathan et al., 2008). B3 TFs are classified into RAV (related to ABI3/VP1), LAV (LEAFY COTYLEDON2-ABI3-VAL), REM (Reproductive meristem), and ARF (Auxin response factor) subfamilies based on domain characteristics and phylogenetic relationships (Swaminathan et al., 2008; Yamasaki et al., 2013). ARF and LAV members contain a single B3 domain whereas REM members possess up to six B3 domains. RAV possesses one B3 domain and an AP2/ERF domain (Swaminathan et al., 2008). The recognition sequence motif 5’-TGTCTC-3’ is an ARF member (Ulmasov et al., 1997) and motifs 5’-CATGCA-3’ and 5’-CACCTG-3’ are LAV members (Ulmasov et al., 1997) and RAV members (Kagaya et al., 1999), respectively. However, the DNA-binding abilities of REM members are still not fully understood and need further investigation (Levy et al., 2002).

*B3* genes are widely involved in plant growth and development. In the LAV group, *Arabidopsis thaliana FUSCA3* regulates seed maturation (Luerssen et al., 1998); maize *ZmABI19* is essential for the initiation of grain filling (Yang et al., 2021); and overexpression of citrus *FUSCA3* promotes somatic embryogenesis (Liu et al., 2018). In the ARF group, overexpression of *AtARF8* affects the development of fruit, hypocotyl and roots (Tian et al., 2004); *AtARF4* regulates the regeneration of shoot meristems (Zhang et al., 2021); and *OsARF8* regulates hypocotyl elongation (Yang et al., 2006). In the RAV group, rice (*Oryza sativa* L.) RAV members regulate flowering time (Osnato et al., 2020), whereas overexpression of strawberry (*Fragaria* × *ananassa*) *FaRAV1* increases anthocyanin production (Zhang et al., 2020). In the REM group, overexpression of Arabidopsis *REM16* accelerates flowering (Yu et al., 2020), and silencing of both *REM34* and *REM35* in Arabidopsis affects the development of reproductive organs (Caselli et al., 2019).

The *B3* genes are also involved in stress and hormone responses. Arabidopsis RAV1 functions in abscisic acid (ABA) signaling by regulating the expression of ABI3, ABI4, and ABI5 in ABA signaling (Feng et al., 2014); whereas overexpression of cotton (*Gossypium hirsutum* L.) *RAV1* in Arabidopsis [*Arabidopsis thaliana* (L.) Heynh.] causes sensitivity to ABA, salt, and drought stresses (Li et al., 2015). Moreover, *AtARF7* is involved in hypocotyl response to auxin (Harper et al., 2000).

B3 TFs have been identified in the genomes of many plant species, including 118 in Arabidopsis, 91 in rice (Swaminathan et al., 2008), 72 in *Citrus sinensis* L. (Liu et al., 2020), 57 in pineapple (*Ananas comosus* L.) (Ruan et al., 2021), 187 in *Brassica rapa* L (Peng and Weselake, 2013), 81 in soybean [*Glycine max* (L.) Merr.] (Peng and Weselake, 2013), and 61 in castor bean (*Ricinus communis* L.) (Wang et al., 2022). However, little is known about Brachypodium [*Brachypodium distachyon* (L.) Beauv.], the model monocot. In this study, we investigated the number, structure, and classification of *B. distachyon B3* TFs. We cloned the gene *BdB3-54* to study its function in root development through overexpression in Arabidopsis. Our study provides a basis for further research on plant *B3* genes.

## Materials and methods

### Identification of B3 TFs

The genome sequences of Brachypodium, Arabidopsis, rice, maize, sorghum (*Sorghum bicolor* L.), barley (*Hordeum vulgare* L.), wheat (*Triticum aestivum* L.), and foxtail millet [*Setaria italica* (L.) Beauv.] were obtained from Ensembl Plants (Bolser et al., 2017). Identification of B3 TFs was carried out in four steps. First, a BLAST search was performed on the obtained genome protein sequences using Arabidopsis and rice B3 protein sequences as queries (threshold: *E*<e^-5^). Second, results from the first step were used to search the B3 structural domain signature model (PF02362) from Pfam (threshold: *E*<e^-5^) (El-Gebali et al., 2019). Third, alternative splicing events and redundancies were manually removed and the NCBI-CDD interface (Marchler-Bauer et al., 2015) was used to confirm putative B3 TFs, removing those without a B3 structural domain.

The physical and chemical properties of B3 TFs were predicted using the ExPASy web server (Artimo et al., 2012), and subcellular localization of B3 proteins was predicted using CELLO (Yu et al., 2004).

### Phylogenetic relationships, gene duplications, and collinearity analyses

MEGA7 software was used to construct the Neighbor-Joining (NJ) trees (Kumar et al., 2016) with 1,000 replications based on the full-length sequence alignment. Segmentally and tandemly duplicated events, and collinearity relationships between BdB3 and other plants were analyzed using MCScanX (Wang et al., 2012). TBtools was utilized to map positions, duplications, and collinearity relationships of the candidate genes (Chen et al., 2020).

### Gene composition analysis

Gene structures were predicted by GSDS 2.0 (Hu et al., 2015). Conserved protein regions were predicted using NCBI-CDD (Marchler-Bauer et al., 2015). Gene compositions were drawn using TBtools (Chen et al., 2020).

### Plant growth, treatment conditions, and RT-qPCR assay

*Brachypodium distachyon* ecotype *Bd21* was grown in an artificial climate chamber under a 16 h light (26°C; 08:00–00:00)/8 h darkness (24°C; 00:00–08:00) cycle. Roots, stems, leaves, young inflorescences, and seeds were sampled 10 d after pollination to determine different tissue expressions. Ten-day-old seedlings were subjected to simulated drought (20% PEG6000), salt (200 mM), heat (42°C), 10 μM 3-indoleacetic acid (IAA), 10 μM salicylic acid (SA), 10 μM ABA, and 10 μM jasmonic acid (JA) treatments for 2 h in hydroponic culture and sampled for different stresses. After sampling, tissues and whole seedlings were collected and stored at -80°C for RNA isolation. Total RNA was extracted using an RNA extraction kit (TIANGEN, Beijing). Next, RT-qPCR was performed in triplicate as previously described (Guo et al., 2021). Relative expression levels were calculated using the 2^–ΔΔCt^ method and normalized to the expression of *BdGAPDH* (Hong et al., 2008) or *AtActin 8* (Reichel et al., 2016).

### Arabidopsis transformation, subcellular localization, and transcriptional assays

The coding sequence (CDS) of *BdB3-54* was amplified by PCR and cloned into *pCambia-1302* and *pCAMV35S-GFP* with a NOS terminator, respectively. *pCambia-1302-BdB3-54* and *pCAMV35S-BdB3-54-GFP* were transferred into the *Agrobacterium tumefaciens* strain *GV3101* through electroporation. Homozygous transformants of Arabidopsis were obtained using the floral dip method (Clough and Bent, 1998). The transgenic lines were screened using hygromycin B solution (40 mg/L) and confirmed by PCR analysis. Third-generation seeds of transgenic lines were used for further analysis. Finally, *pCAMV35S-BdB3-54-GFP* was transformed into tobacco (*Nicotiana tabacum* L.) leaves using the *GV3101* strain for subcellular localization with an Olympus IX83-FV1200 confocal microscope (Olympus, Tokyo).

The *pGBKT7-BdB3-54* vector was constructed for yeast autoactivation assays. Then, *pGBKT7*-*BdB3-54*, negative vector *pGBKT7*, and positive vector *pGBKT7*-*p53* were transformed into yeast strain Y2H. The surviving clones were grown on SD/-Trp medium, and the transformed yeast cells were diluted and dotted on SD/-Trp and SD/-Trp/-Ade/-His media. Cells were incubated at 30°C for 3 d. Primers used in this study (**Supplementary Table S1**) were designed using the Oligo 7 software (Rychlik, 2007).

### Phenotypic observations and statistical analyses

Arabidopsis seedlings were grown in a growth chamber under a 16 h light (22°C; 08:00–00:00)/8 h darkness (20°C; 00:00–08:00) regime. Root length was measured on the tenth day and counted using the ImageJ software (Rueden et al., 2017). Photos of the root apical meristem cell on the 4-day-old plants were taken after staining with propidium iodide (PI, 0.01 mg/ml) for 1-2 min using confocal microscopy (Olympus IX83-FV1200, Japan) with a 561-nm laser for PI. Data were analyzed and plotted using the IBM SPSS Statistics software (USA). Values are shown as means ± SD, and significant differences are indicated by different letters or e-values (*P <*0.05, one-way ANOVA).

## Results

### B3 TFs in six monocot plants

A comprehensive search of the six monocot plant species identified 69, 589, 99, 92, 130, and 91 *B3* genes in *B. distachyon*, wheat, maize, foxtail millet, barley, and sorghum, respectively (**Table 1**). Based on the characteristics of the conserved domains and the number of B3 domains, these genes were classified into four subfamilies: 250 in ARF, 76 in RAV, 890 in REM, and 63 in LAV (**Supplementary Tables 2, 3**).

**Table 1:** Numbers of *B3* genes identified in different plant species.

Plant species	ARF	RAV	REM	LAV	Total	Proportion of the genome (%)
*Brachypodium distachyon*	24	4	36	5	69	0.20
*Oryza sativa^#^*	28	16	40	7	91	0.24
*Triticum aestivum*	66	26	479	18	589	0.55
*Zea mays*	39	5	47	8	99	0.25
*Setaria italica*	24	6	57	5	92	0.26
*Hordeum vulgare*	21	3	99	7	130	0.36
*Sorghum bicolor*	25	3	56	7	91	0.27
*Arabidopsis thaliana^#^*	23	13	76	6	118	0.43
Total	250	76	890	63	1279	

^#^B3 members in Oryza sativa and Arabidopsis thaliana were reported in Swaminathan et al. (2008) research.

### B3 TFs in Brachypodium distachyon

**Figure 1:**
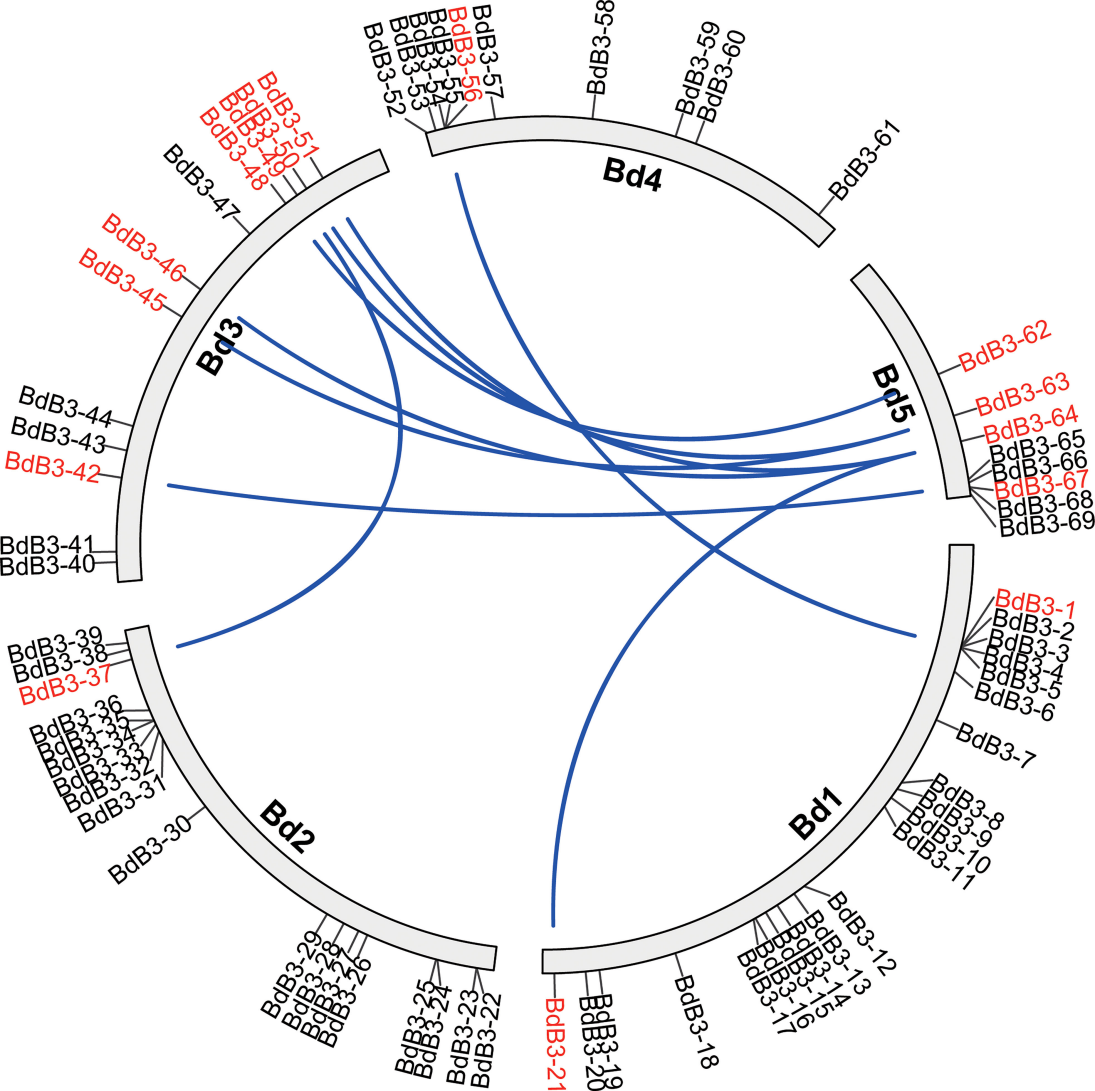
Chromosome location and segmental duplication of the *BdB3* genes. Blue lines connect duplicated genes that are shown in red.

The 69 putative B3 TFs were unevenly distributed on five *B. distachyon* chromosomes with 21, 18, 12, 10, and 8 on chromosomes 1, 2, 3, 4, and 5, respectively (**Figure 1**). They were named *BdB3-1* to *BdB3-69* and validated with expressed sequence tags (ESTs) from the NCBI database. The predicted length of the BdB3 proteins ranged from 166 (BdB3-16) to 1,227 (BdB3-55) amino acids with molecular weights ranging from 18.10 (BdB3-16) to 139.50 (BdB3-55) kDa, and the genomic sequence lengths ranged from 1,837 bp (*BdB3-22*) to 14,692 bp (*BdB3-55*) (**Supplementary Table 3**). Protein subcellular localization prediction showed that 62 BdB3 proteins were localized in the nucleus, three in the cytoplasmic, three in the chloroplast, and one (BdB3-68) in the extracellular matrix.

### Synteny and homologous gene pairs

**Figure 2:**
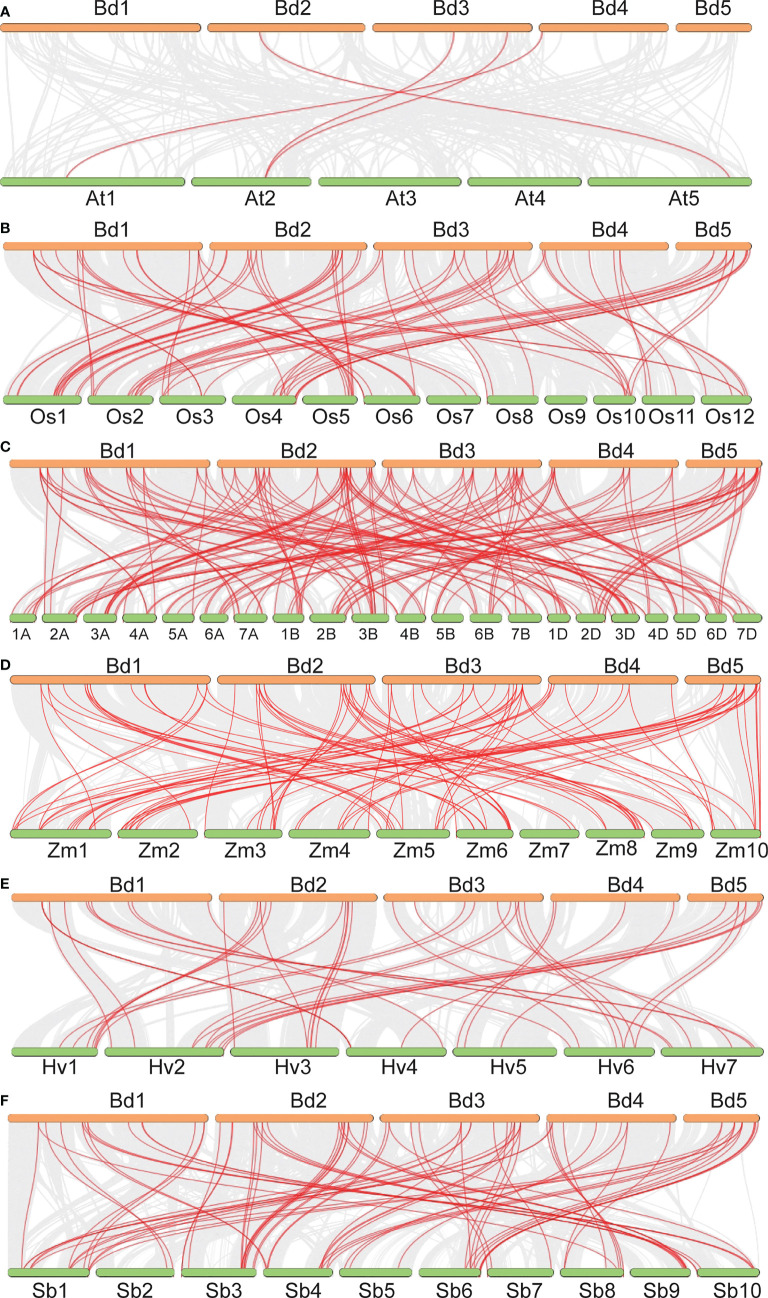
Synteny analysis of the *B3* genes between *B distachyon* and *A thaliana* **(A)**, *O. sativa* **(B)**, *T. aestivum* **(C)**, *Z. mays* **(D)**, *H vulgare* **(E)**, and *S. bicolor* **(F)**. Gray lines in the background indicate the collinear blocks within *B distachyon* and other plant species, and red lines highlight syntenic *B3* gene pairs.

Gene duplication analysis detected 15 tandemly duplicated genes. They formed nine gene pairs. Among them, *BdB3-64* was pared with three genes (*BdB3-21, BdB3-46*, and *BdB3-51*) and *BdB3-63* was pared with two genes (*BdB3-45*, and *BdB3-50*) (**Figure 1**, **Supplementary Table 4**). Genome synteny between *B. distachyon* and the other plant species showed 3, 51, 52, 41, 52, and 47 *BdB3* gene homologs in Arabidopsis, rice, wheat, barley, sorghum, and maize, respectively (**Figure 2**, **Supplementary Table 5**). These results suggest that *BdB3* genes share similar structures and functions with orthologs in other monocot plants.

### Phylogenetic trees and gene components of BdB3 TFs

**Figure 3:**
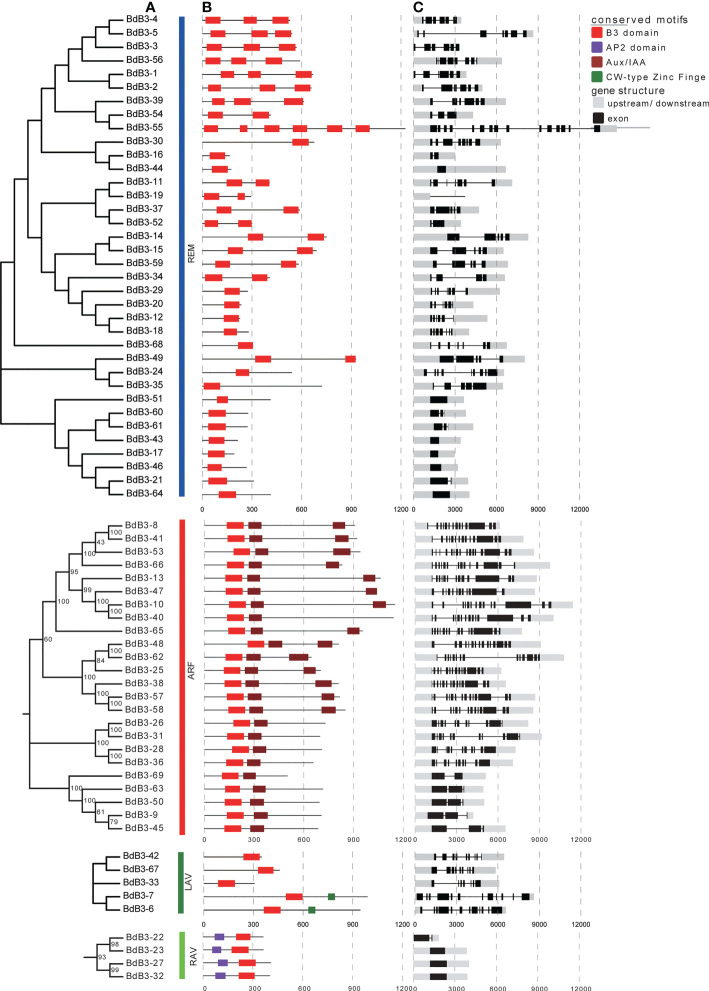
Phylogenetic relationships, conserved protein domains, and gene structures of *B distachyon* REM, RAV, ARF, and LAV members. **(A)** Neighbor-joining trees constructed for *B3* genes from the four subfamilies. **(B)** The four conserved protein domains are shown in different colors. **(C)** Structures of *BdB3* genes. Gray boxes indicate up- or down-stream structures, black boxes indicate exons, and black lines indicate introns.

NJ trees for the four subfamilies were constructed according to sequence characteristics to explore the phylogenetic relationships of BdB3 TFs (**Figure 3A**). As shown in **Figure 3B**, each B3 member contained at least one B3 domain; REM members contained 1 to 6 B3 domains; and ARF members contained a single B3 domain at the N-terminus and one or two Aux/IAA domains (carboxyl-terminal interaction domains). LAV members had one B3 domain at the C-terminus and two members (BdB3-6 and BdB3-7) had a CW-type zinc finger. Each RAV member contained one AP2 domain at the N-terminus and one B3 domain at the C-terminus.

Exon numbers in *B3* genes ranged from one to 16 (**Figure 3C**). Each subgroup had a different number of exons; RAV members contained 1-2 exons, whereas the REM members had 1-15. Additionally, 22 members contained more than 5 exons, and all LAV members had more than 7 exons. The exon number in ARF members varied greatly with five having 2 or 3 exons, and the other members having more than 10 exons. This structural diversity implies diverse functions for *BdB3* genes.

### Expression pattern analyses

**Figure 4:**
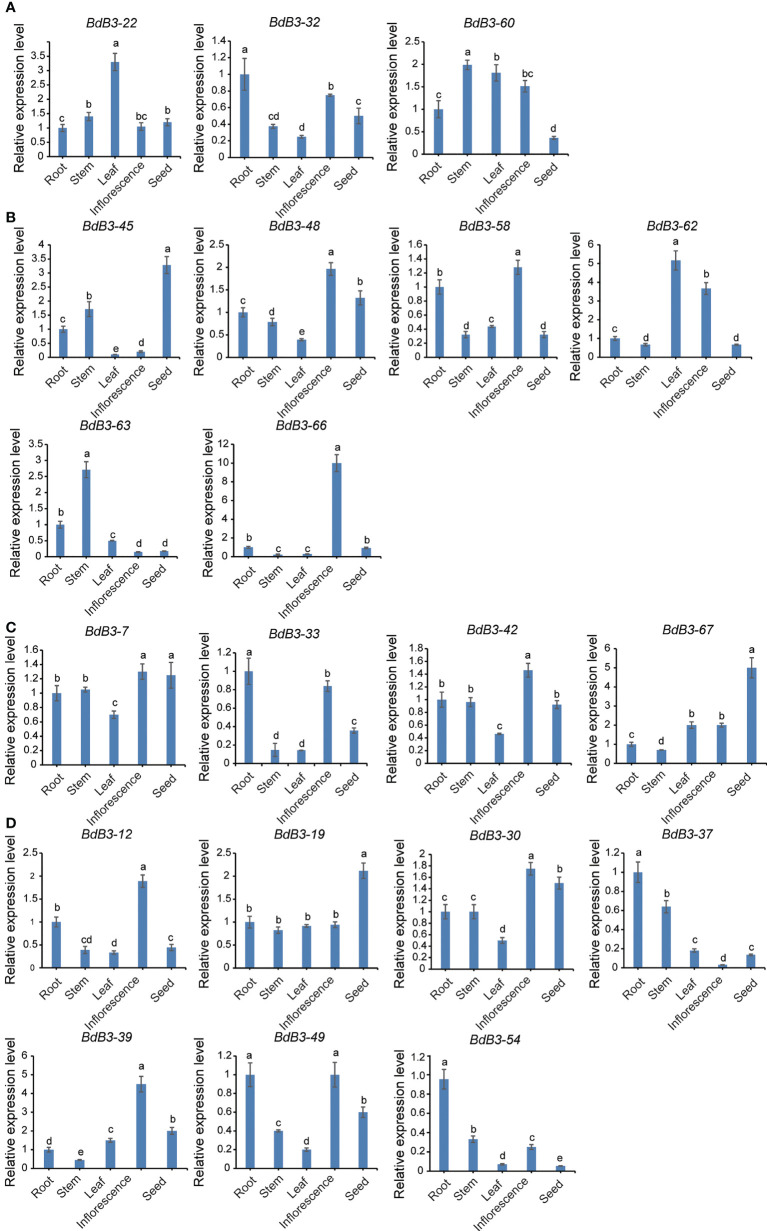
Expression patterns of *BdB3* genes in different tissues. **(A–D)** represent the expression patterns of RAV, ARF, LAV, and REM genes. Abscissas represent different tissues, including roots, stems, leaves, early inflorescences, and seeds at 10 days after pollination. Ordinates represent relative expression levels. Transcript levels of *BdB3* genes were normalized to those of *BdGAPDH*, and expression levels of root tissues were set to 1. Data are shown as means ± SE (n = 3) Letters above the bars indicate significant differences (P <0.05, one-way ANOVA)..

Twenty *BdB3* genes, including 3 RAV, 6 ARF, 4 LAV, and 7 REM members, were analyzed for expressions levels in different tissues (roots, stems, leaves, young inflorescences, and seeds sampled 10 days after pollination) using RT-qPCR. Expression of these *BdB3* genes was detected in all tissues (**Figure 4**, **Figure S1A**). For example, the LAV genes *BdB3*-*7* and *BdB3*-*67* were highly expressed in seeds, whereas *BdB3*-*33* and *BdB3-42* were highly expressed in roots and inflorescences, respectively. Further, the REM genes *BdB3-12*, -*30*, -*39*, and -*49* had high expression levels in inflorescences, whereas *BdB3-37*, -*49*, and -*54* showed high expression levels in roots.

**Figure 5:**
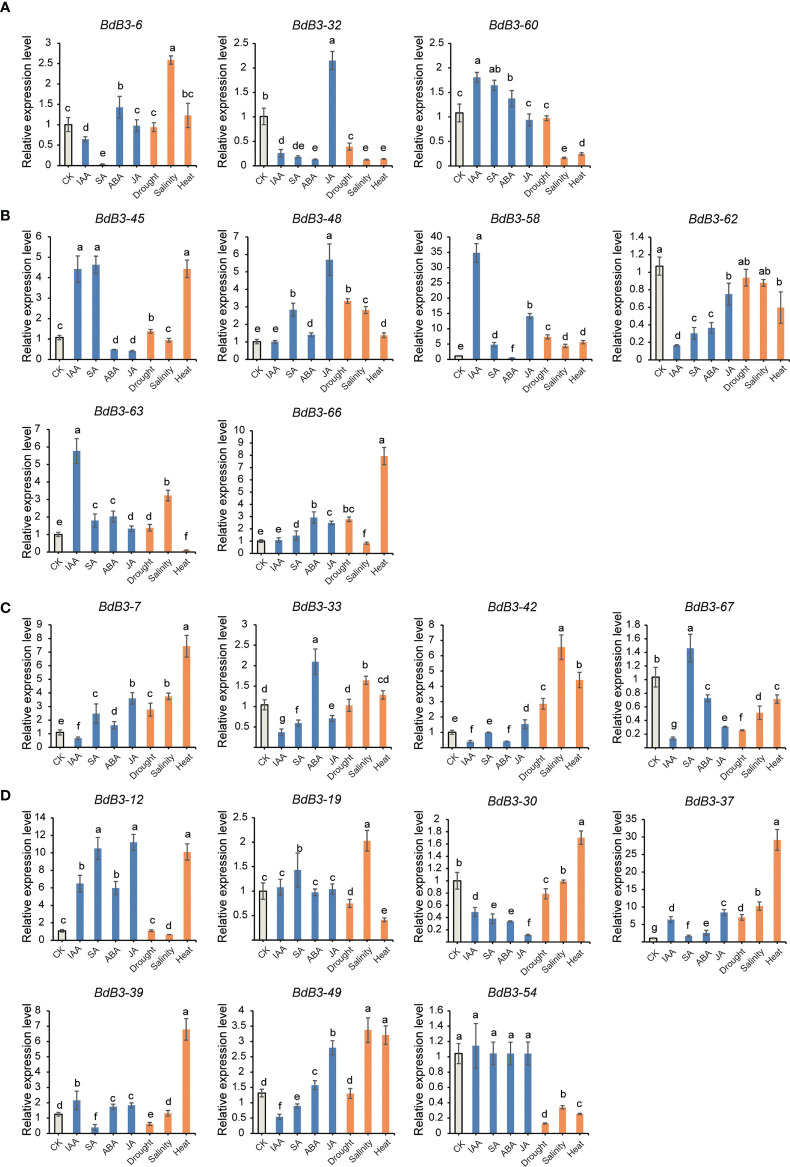
Expression patterns of *BdB3* genes under different hormonal and abiotic stress conditions. **(A**–**D)** represent the expression patterns of RAV, ARF, LAV, and REM members. Abscissas represents different stress of hormone treatments. CK, non-treated. Ordinates represent the relative expression levels. Data are means ± SE (n = 3). Letters above the bars indicate significant differences (*P* <0.05, one-way ANOVA).

The expression of selected *BdB3* genes under abiotic and hormonal stresses varied considerably compared to the control (no treatment) (**Figure 5**, **Figures S1B, C**). For the RAV family, genes *BdB3-22* and *BdB3*-*32* were significantly down-regulated by IAA and SA, and *BdB3-22* and *BdB3*-*32* were significantly down-regulated by salinity and heat. JA had the greatest impact on the expression of *BdB3*-*32*, whereas *BdB3-60* was up-regulated by IAA, SA, and ABA. Three (*BdB3-45*, *-58*, and *-63*) and five (*BdB3-45*, *-48*, *-58*, *-63*, and *-66*) ARF genes were highly expressed under IAA and SA treatments, respectively. Five genes (*BdB3-45*, *-48*, *-58*, -*63*, *-66*), four genes (*BdB3-45*, *-48*, *-58*, and *-66*), and three genes (*BdB3-48*, *-58*, and *-63*) were up-regulated under drought, heat, and salinity stresses. *BdB3-62* was down-regulated by all stress conditions. All LAV genes were down-regulated by IAA. *BdB3-7* was up-regulated under all different treatments apart from IAA. Among REM genes, heat stress had the greatest impact on their expressions with *BdB3-12*, *-30*, *-37*, *-39*, and *-49* being up-regulated and genes *BdB3-19* and -*54* being down-regulated. Hormones including IAA, SA, ABA, and JA significantly regulated the expressions of *BdB3-12*, *-30*, *-37*, *-39*, and *-49*.

### Subcellular localization and transactivation assay of BdB3-54

**Figure 6:**
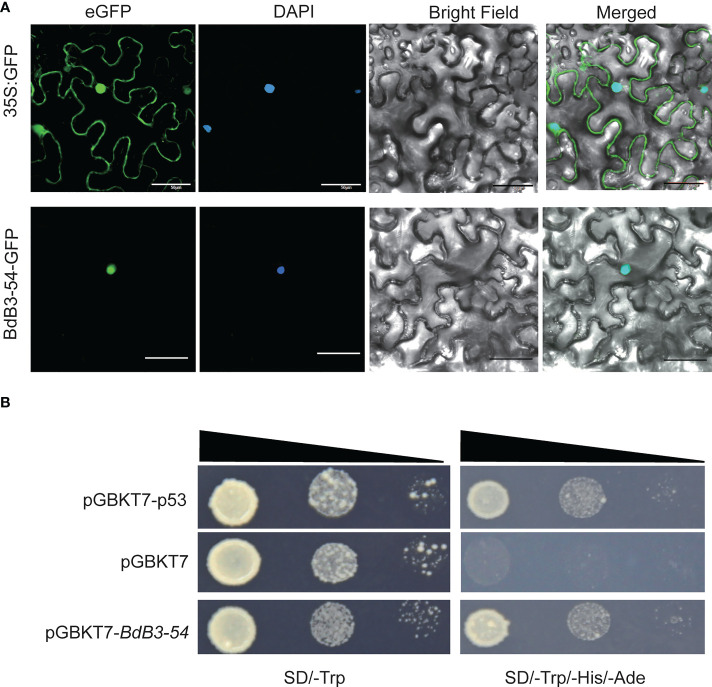
Subcellular localization and transactivation assay of BdB3-54. **(A)**. BdB3-54 protein was transiently expressed in tobacco (*Nicotiana benthamiana* L.) leaves to determine its subcellular localization; bar, 50 μm. **(B)**. BdB3-54 transactivation assay. BdB3-54 was ligated to the pGBKT7 vector, and transformed yeast cells were screened on SD/-Trp and SD/-Trp-His-Ade media.

*BdB3-54* belongs to the REM subfamily and its functions have rarely been investigated. Subcellular localization analysis predicted that BdB3-54 protein was localized in the nucleus. When transiently expressed in tobacco leaves, *BdB3-54* fusion protein signals overlapped the DAPI signal confirming that BdB3-54 protein was located in the nucleus (**Figure 6A**).

A transactivation assay was performed to test the transcriptional activation activity of *BdB3-54* using Y2H assays. Yeast cells carrying the *pGBKT7-BdB3-54* plasmid grew well on the defective SD/-Trp-His-Ade medium, which was similar to *pGABKT7-p53*, a positive control plasmid. In contrast, yeast cells carrying the negative control *pGBKT-7* showed much less growth (**Figure 6B**). These results indicated that *BdB3-54* had transcriptional self-activation activity.

### Overexpression of *BdB3-54* in Arabidopsis increased primary root length

**Figure 7:**
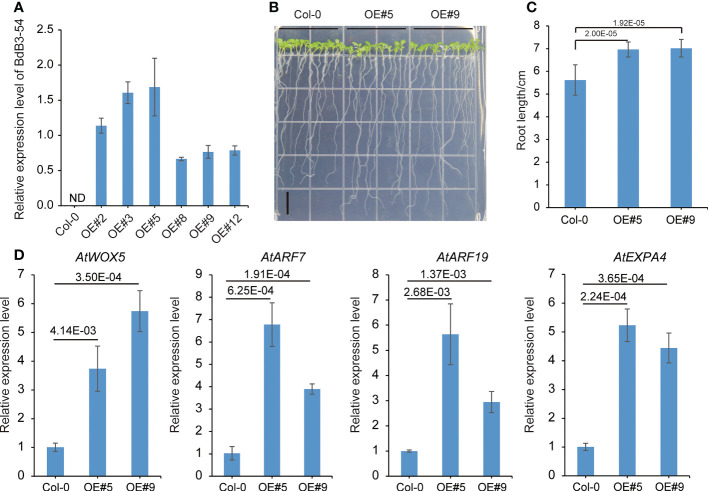
Root phenotypes of Arabidopsis lines overexpressing *BdB3-54*. **(A)** Relative expression levels of *BdB3-54* in six transgenic T_3_ lines. ND, expression not detected. **(B)** Phenotypes of Arabidopsis lines overexpressing *BdB3-54* (10-day-old plants). Scale bar, 1 cm. **(C)** Statistical results of root length. **(D)** Relative expression levels of four root-development genes in Arabidopsis. Data are means ± SE (n = 3). Numbers above the horizontal line indicate the significance of differences (one-way ANOVA).

Expression pattern analysis showed that *BdB3-54* was highly expressed in root tissues (**Figure 4D**). To determine its role in root development, two transgenic Arabidopsis lines overexpressing *BdB3-54* driven by the CaMV35S promoter were generated (**Figure 7A**). The primary root lengths of transgenic plants overexpressing *BdB3-54* were significantly longer than the wild-type (WT) Col-0 (**Figures 7B, C**). To explore potential factors leading to the longer primary root lengths of transgenic plants, we examined the root apical meristem cell size on the 4-day-old plants. With the same number of cells, the transgenic lines occupied a larger area (**Figure S2**). We also investigated the gene expression of four root development-related genes in the WT and the transgenic lines *AtWOX5*, *AtARF7*, *AtARF19*, and *AtEXPA4*. As shown in **Figure 7D**, the expression levels of these genes were significantly higher in transgenic plants than in the WT (*P <*0.05). These results indicated that ectopic expression of *BdB3-54* regulated primary root growth in Arabidopsis.

## Discussion

### Characteristics of B3 TFs in *B. distachyon*

We identified 69 B3 genes in the *B. distachyon* genome. The proportion of *B3* genes in the *B. distachyon* genome was approximately 0.20%, which was less than that in rice (0.24%), wheat (0.38%), Arabidopsis (0.43%), and other monocot species (**Table 1**) (Swaminathan et al., 2008). The gene number of three subfamilies, excluding ARF members, was lower than that in other plant species, suggesting that gene loss had occurred during evolution.

The *B3* genes in *B. distachyon* were grouped into REM, ARF, LAV, and RAV subfamilies according to protein characteristics and the number of B3 domains. Each subfamily member shares a similar domain composition and gene structure. Gene structures for different subfamilies showed significant variation in intron number and length, indicating that these *B3* genes might have undergone intron loss or gain during evolution. Phylogenetic analysis of different subfamilies in previous studies indicated that the same clade members also shared similar gene components, including gene structure and conserved domains, suggesting conserved functions and common origins (Bhattacharjee et al., 2015; Verma and Bhatia, 2019).

### Diverse functions of B3 TFs

Genes perform functions according to their expression in different tissues, and gene expression patterns reflect the gene function. For example, many Arabidopsis *B3* genes, such as *ARF3*, *ARF5*, *ARF6*, *ARF8*, and *ARF9*, have diverse functions in the development of carpels, floral parts, and fruit, as well as lateral roots (Li et al., 2016; Zhang et al., 2018). AtVAL-1, -2, and -3 proteins are required for seed germination (Suzuki et al., 2007; Jia et al., 2013; Schneider et al., 2016). Tissue-specific expression patterns in the present study indicated that *BdB3* genes were expressed in all tissues examined, and members in different subfamilies showed different expression patterns, further indicating their functional diversity in plant growth and development. Genome-wide expression pattern analyses showed that wheat, citrus, and cotton *B3* genes also had different expression patterns in different tissues (Liu and Zhang, 2017; Liu et al., 2020; Luo et al., 2022).

*BdB3* genes displayed significant differential expression under different abiotic stress and hormone conditions, suggesting crucial roles in response to stress. Hormones such as IAA, ABA, SA, and JA are known to regulate stress-related pathways as plants grow and develop (Verma et al., 2016). Most *BdB3* genes responded differentially under different hormones, suggesting that hormones specifically regulated the expression of *BdB3* genes under certain conditions. Similarly, hormones also regulate the expression of plant *B3* genes in other species, such as Arabidopsis, chickpea, and citrus, indicating that the functions of *B3* genes are diverse but conserved across plant species (Verma and Bhatia, 2019; Liu et al., 2020).

### *BdB3-54* functions as a TF and has a key role in root development

Various studies have reported that *B3* genes are involved in plant root growth and development. For examples, the LAV member FUSCA3 interacts with LEC2 to control the formation of lateral roots in Arabidopsis (Tang et al., 2017); RAV member GmRAV1 is an important positive regulator involved in promoting root regeneration in Arabidopsis and soybean (Zhang et al., 2019); Ectopic expression of *TaARF4-A* in Arabidopsis leads to shortened primary root length (Wang et al., 2019); *AtARF7* and *AtARF19* regulate the formation of lateral roots through the activation of *LBD/ASL* genes (Okushima et al., 2007). Our study showed that *BdB3*-*54* contained two B3 domains and acted as a TF in *B. distachyon*, which is supported by the fact that a *BdB3*-*54*-GFP fusion protein was localized in the nucleus and had transcriptional activity in yeast cells.

*BdB3-54* was highly expressed in root tissue, and overexpression of the gene increased root length when compared to the WT. These observations suggest that the *B3* genes are involved in root development and growth, and the longer root length in transgenic plants is mainly due to enlarged cells. Root development-related genes, such as expansins (*EXP*), WUS-related homeobox genes (*WOX*s), and *ARF*s, were also detected during our investigation of *BdB3-54* function. Among these root development-related genes, *AtWOX5* is expressed in the quiescent center and affects root development (Kong et al., 2015); *AtARF7* and *AtARF19* regulate the formation of lateral root formation *via* direct activation of the downstream genes (Okushima et al., 2007); and *AtEXPA4* is involved in root elongation (Liu et al., 2021). Expression of *AtWOX5*, *AtARF7*, *AtARF19*, and *AtEXPA4* was up-regulated in a transgenic Arabidopsis line carrying *BdB3-54* suggesting that *BdB3-54* regulates the expression of other root development-related genes. *B. distachyon*, wheat, and rice all belong to pooideae. The function of *BdB3-54* in root development indicated that it can be used for molecular breeding in cereal crops

In conclusion, 69 *B3* genes were identified in the *B. distachyon* genome. These genes were expressed in different plant tissues and showed different responses to various stresses. Further study on one of the highly expressed genes, *BdB3-54*, indicated that this gene functions as a TF and has an important role in root development.

## Data availability statement

The original contributions presented in the study are included in the article/**Supplementary Material**. Further inquiries can be directed to the corresponding authors.

## Author contributions

JG analyzed the data and wrote the manuscript. HL and KD helped to carry out the experiments. PG and XY contributed to writing the manuscript. MZ and WS contributed to experimental design, provided advice for data analysis, and assisted in writing the manuscript. All authors contributed to the article and approved the submitted version.

## Funding

This work was supported by grants from the National Key R&D Program of China (2021YFD1901101), the Key Science and Technology Program of Shanxi Province, China (20210140601026), the National Natural Science Foundation of China (31901541) and the Project of Biological Breeding of Shanxi Agricultural University, China (YZGC102). The funding body did not exert influence on the design of the study, collection, analysis, and interpretation of data, or in writing the manuscript.

## Acknowledgments

We gratefully acknowledge help from Professor Hongjie Li (Chinese Academy of Agricultural Sciences) and Robert A. McIntosh (University of Sydney), with English editing.

## Conflict of interest

The authors declare that the research was conducted in the absence of any commercial or financial commitments that could be construed as a potential conflict of interest.

## Publisher’s note

All claims expressed in this article are solely those of the authors and do not necessarily represent those of their affiliated organizations, or those of the publisher, the editors and the reviewers. Any product that may be evaluated in this article, or claim that may be made by its manufacturer, is not guaranteed or endorsed by the publisher.
