# Supplementary material for: Identification of Brachypodium distachyon B3 genes reveals that BdB3-54 regulates primary root growth
Source: Front Plant Sci. 2022 Nov 10;13:1050171. doi: 10.3389/fpls.2022.1050171 (PMC9686306; doi:10.3389/fpls.2022.1050171)
Supplement: Supplementary file 2 [file DataSheet_2.pdf]

Supplementary Figures

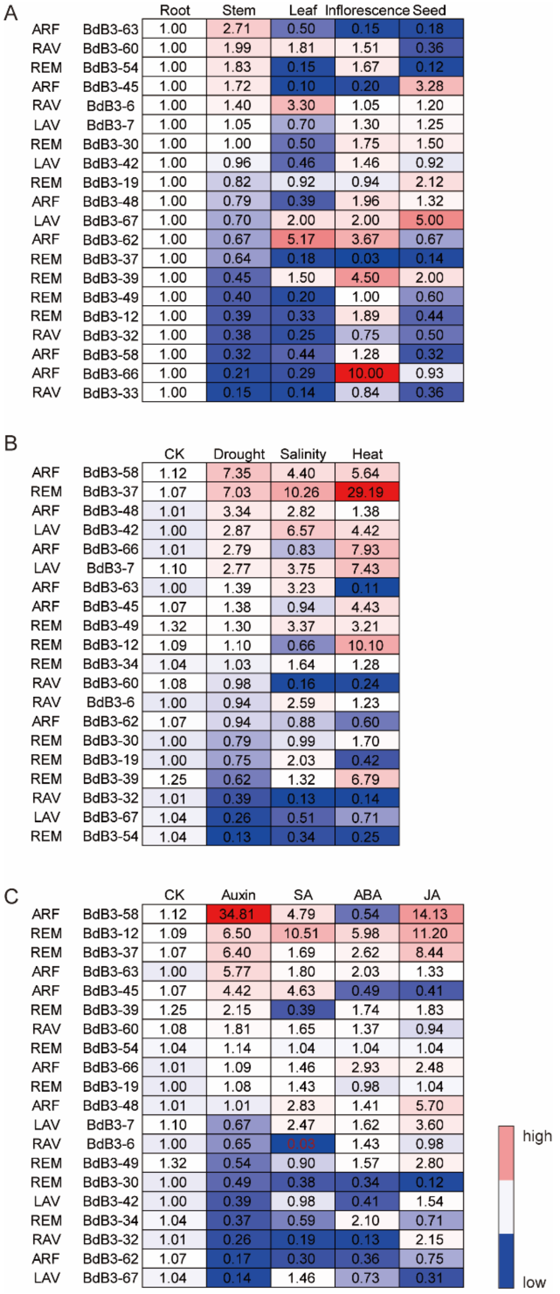

Figure S1 Gene expression heat maps of *BdB3* genes in different tissues (A), under different abiotic stress (B) and hormonal (C) conditions. Data are the mean average value of the expression (n = 3). The color represents expression levels, the redder the expression is, the higher the expression is; the bluer the expression is, the lower the expression is.

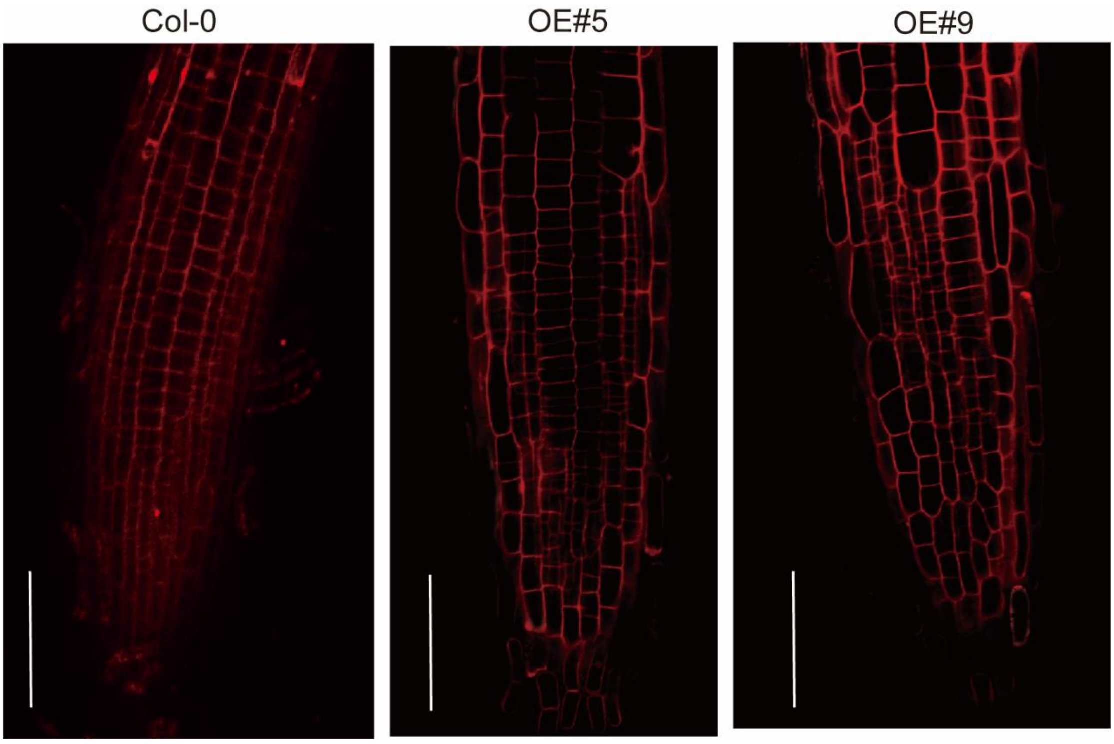

Figure S2 Confocal images of root tips of Col-0 and transgenic lines. 4-day-old seedlings of Col-0 and transgenic lines were stained with 10 mg/μl propidium iodide (PI) for 1-2 min, and then observed by CLSM. Scale bars = 100 μm.
